# Institutional logics as an object of change: the experiences of a water organization using design thinking for climate adaptation in a multi-stakeholder process

**DOI:** 10.1007/s11625-025-01660-4

**Published:** 2025-03-24

**Authors:** Florian Goldschmeding, René Kemp, Véronique Vasseur, Christian Scholl

**Affiliations:** 1 Maastricht Sustainability Institute, School of Business and Economics, Maastricht University, Tapijn 11 Building D, P.O. Box 616, 6200 MD Maastricht, The Netherlands; 2 United Nations University-Maastricht Economic and Social Research Institute on Innovation and Technology (UNU-MERIT), United Nations University, 6211 AX Maastricht, The Netherlands

**Keywords:** Institutional logics, Design thinking, Water management, Dutch water sector, Institutional change

## Abstract

In the search for solutions to complex challenges posed by climate change and sustainability transitions, organizations often turn to innovative approaches and new cognitive frames. Particularly in the public sector, however, entrenched institutional logics often impede progress toward novel solutions. This paper explores how a public organization in the Dutch water sector navigates competing and sometimes conflicting frames and institutional logics through design-thinking. The object of study is a 6-month project initiated by a regional water authority in response to a severe flooding in 2021. The project aimed to develop and reimagine instruments for inclusive and climate-adaptive water management in collaboration with diverse actors. Based on participant observation and qualitative interviews, we analyze a series of design-thinking workshops where diverse stakeholders co-created tools for climate-adaptive water management. The findings disclose the existence of significant conflicts between the frames and institutional logics and offer details on how these were addressed through repeated stakeholder interaction and institutional work. We found that individuals showed varied responses to the emerging institutional logics, and that dominant institutional logics were diversely interpreted by different actors within the organization. Our research shows how iterative, participatory design methods can help actors temporarily shift institutional logics, but also reveals persistent challenges in achieving enduring changes to dominant institutions.

## Introduction

Climate change and sustainability transitions present societal actors with complex problems that often require innovative and integrated solutions, as well as new ways of thinking and working (Diepenmaat et al. 2020). New ways of thinking (cognitive schemes) are examples of institutional change, which may conflict with existing institutional logics (Fuenfschilling and Truffer 2014). Institutional logics are the beliefs, norms, and rules that govern behavior in organizations or societal sectors (Thornton and Ocasio 1999). Complex problems can reveal tension between diverse institutional logics that govern the decision-making and operations in various sectors. One sector where tensions between institutional logics are particularly salient is the water sector (Fuenfschilling and Truffer 2014). In a time of climate change and assertive citizens, (public) organizations are pressured to become more sustainable, more democratic and deal with the consequences of climate change in the form of droughts and flood risks, but also to keep the costs of water management as low as possible. This has produced a situation where conflicts between existing institutional logics emerge and need to be resolved to produce coherent strategic and operational frames of action (Franco-Torres et al. 2021).

In transition studies and sustainability science, design thinking has emerged as a promising approach for addressing complex sustainability problems, by opening up problem and solution spaces, and by incorporating diverse perspectives into the development of solutions (Leal Filho et al. 2024; Simon 2023). In the enthusiasm about design, both practitioners and academics may overlook that […] “designing unfolds in a world that is already interpreted, where people are already acting […] for reasons that are taken-for-granted. These taken-for-granted reasons are lost in history and hard to retrieve, if retrieval were even an issue. The question “why are we doing this” seldom comes up in the mood of thrownness because acting with what is at hand is primary and detached reflection secondary” (Weick 2004). By focusing on the creative element, the influence of dominant frames and institutional logics that may restrict the creative space is easily missed or overlooked.

This paper describes the experiences with design thinking in a (public) water organization in the Netherlands, Waterschap Limburg, which faces increasing challenges to deal with climate change consequences such as flood risks and droughts (Waterschap Limburg 2023), and is in increased interaction with regional governments, citizens, and other stakeholders. The project studied in this paper was sparked by a dramatic flooding event in July 2021, in the region where, following excessive rain in over 48 h, many villages in Germany, The Netherlands, and Belgium were flooded and severely damaged, resulting in over 200 lives lost (Hagenlocher et al. 2023; Mahtani et al. 2021). The flooding revealed that the towns located in (narrow) river valleys were poorly protected against sudden heavy rainfall and signaled a need for better protection and international collaboration (Hagenlocher et al. 2023). The design project, named ‘Climate-Adaptive Registration’ (CARE) was initiated by Waterschap Limburg (WL), a public organization engaged in water management, as part of an international research project (INTERREG EMFloodResilience), established to support research on climate resilience. This project included 11 partners from the regions strongly affected by the flooding in Belgium, Germany, and the Netherlands. Within the CARE subproject, WL opted for a design-based approach to develop new practices and instruments that support climate-adaptive water management.

In exploring the institutional dynamics at play within the project, institutional theory provides an analytical lens to understand how institutions (regulative, normative, cognitive) shape (constrain and enable) organizational behavior (Powell and Bromley 2015). The concept of institutional logics, as systems of cultural elements and practices embedded within organizations, is particularly relevant, in drawing attention to the existence of competing logics and the need for reconciling (combining) these in the pursuit of new problem-solving approaches. For instance, the water authority operates according to technical logic for landscape interventions (assets), community logic for engaging stakeholders, and efficiency logic for managing taxation or budget decisions. While literature often views such logics as discrete and explicit (e.g., Fuenfschilling and Truffer 2014; Thornton et al. 2012), we find that, in practice, they rather appear contingent on situational factors and latent (becoming manifest through specific actions).

The evolutionary perspective on institutions regards them as dynamic and under continuous development, in part because actors within organizations can engage in deliberate reflexive actions to maintain, tinker with, or disrupt entrenched practices and norms through a process of institutional work (Lawrence et al. 2009). Institutional work can manifest as acts of framing and reframing that can be used to challenge extant frames. However, the literature on institutional logics and institutional work often focuses on actors within organizations, overlooking the impact of external developments, actors, and collaborative efforts beyond organizational boundaries. Additionally, design thinking is increasingly discussed as a useful method for achieving transformative innovation (Leal Filho et al. 2024; Simon 2023; Irwin 2018), though its potential to support institutional work and manage conflicting institutional logics remains unexplored. The present paper studies whether and if so how external collaborations and innovative methodologies can support organizations in managing complex institutional dynamics. A unique aspect of the project is that the first author was involved in the design process as a participant observer, with deep knowledge of the organization obtained over 4 years of work as an embedded action researcher. The first author was embedded within the host organization, performing a range of research (e.g., observation, feedback) and functional tasks (e.g., advisory, project management), while, on average, spending 3 days per week at the host organization.

Focusing on the CARE project as a case study, this research studies how features of the design-thinking project facilitated engagement with diverse logics within a protected setting, enabling temporary release of dominant logics or frames, and fostering institutional work. It responds to the call from Thornton et al. (2012) for research on the mechanisms and implications of logic recombination, and the call by Fuenfschilling and Truffer (2014) for investigating forms of institutional work used to alter or maintain institutional settings. Scientifically, it is part of an expanding research line within contemporary institutional research on “how institutions and broader institutional fields are constructed, sustained and altered in micro-(political) struggles over frames and their consequences” (Werner and Cornelissen 2014).

The remainder of this paper is structured as follows: “Theoretical background” provides a concise overview of the literature on (conflicting) institutional logics and institutional work, frames, and framing, forming a conceptual framework for examining the case study. “Project context and description/case study overview” provides a brief overview of the case investigated and the project’s format. “Methodology” describes the methodology employed and the data collected. “Navigating diverse institutional logics in the CARE project” presents a detailed analysis of the events of the studied case and provides key findings on ‘managing’ (conflicting) institutional logics through design-thinking, and it discusses to what extent the design-thinking project facilitated engagement with dominant logics and enabled institutional work, as well as the responses of organization members to the introduction of new logics. “Discussion” links our findings to concepts from the literature on frames, institutional logics, and institutional work, highlighting the implications of the research findings for theory and practice in organizational studies and water management. “Conclusion” summarizes our findings, presents final conclusions, and suggests avenues for future research.

## Theoretical background

To investigate the process by which organizations adapt to changing requirements and societal trends, it is useful to draw on the field of institutional theory. Institutional theory provides a framework for understanding how institutions (e.g., norms, rules, beliefs) influence the behaviors and practices of organizations (Lawrence et al. 2009). Such norms, rules, and beliefs impact how an organization performs its regular operations, but also influence organizational responses to unforeseen shocks, external demands, or emerging developments.

A useful perspective within institutional theory is the evolutionary institutional perspective, since a common criticism of institutional analysis is its rather static and deterministic view (Purdy et al. 2019). This approach, however, emphasizes the emergent and gradual nature of institutional change, characterizing institutions as dynamic and adaptive (Kingston and Caballero 2009; Lewis and Steinmo 2012). In this perspective, institutions gradually change over time due to the influence of internal and external pressures. Specifically, it highlights how institutions are not static, but rather under continuous (iterative) development in varying directions (Kingston and Caballero 2009). The evolutionary lens applied to our case helps us understand how WL and its institutions are continually adapting to internal pressures and changing external circumstances, societal demands, and changes in the physical environment. Several relevant elements of institutional theory are introduced below, followed by a brief discussion of design thinking as an approach to solving complex problems, providing a comprehensive theoretical basis for the remainder of the paper.

### Institutional logics

As a central concept in institutional theory, institutional logics refer to the rules, norms, and beliefs that shape the thinking and evaluation of individuals who adhere to it (Thornton and Ocasio 1999). Institutional logics are the link between institutional orders and action. They involve schemes that guide the actions of individuals within organizations by shaping how they understand and respond to various situations and define criteria for decision-making. For example, a market logic prioritizes efficiency and competition, while a professional logic emphasizes procedure and compliance.

Friedland and Alford (1991, p. 248) defined institutional logics as “a set of material practices and symbolic constructions [that] constitute organizing principles” for “patterns of human activity”. A broader definition of institutional logics is offered by Thornton and Ocasio (1999, p. 804) for whom institutional logics comprise “the socially constructed, historical patterns of material practices, assumptions, values, beliefs, and rules by which individuals produce and reproduce their material subsistence, organize time and space, and provide meaning to their social reality”. A multitude of elements are included, e.g., ‘assumptions’, ‘rules’, ‘values’, ‘beliefs’ and ‘time’, which does not make it very sharp: “the definition lost in analytical operationalizability what it gained in breadth” (Berg Johansen and Waldorff 2017). Arguably, the same can be said for the concept of socio-technical regime used in transition studies (Fuenfschilling and Truffer 2014; Geels 2020).

Both institutional logics and socio-technical regimes can be viewed as frameworks that guide and constrain the actions of actors within a particular field or domain. Just as institutional logics comprise rules, assumptions, and beliefs that guide organizational behaviors, socio-technical regimes represent embedded structures and rules that govern the decision-making and problem-solving actions of actors within a particular technological system or industry (Rip and Kemp 1998; Geels and Schot 2007). In both cases, these frameworks represent a certain stability and continuity, while at the same time being subject to changes, particularly when confronted with external pressures or innovations. Institutional logics and socio-technical regimes are conceptually different but can offer complementary perspectives on stability and change in the context of the water sector. Where institutional logics operate on a localized, intangible level, embedded within organizations, socio-technical regimes are broader in scope and contain both intangible and material elements (i.e., infrastructure, technologies). Still, both are frameworks that shape how actors understand and engage with their environment, how they make decisions, and that define the context of their practices. Ultimately, they are complementary lenses through which to view the interactions between actors and the changing systems of which they are part.

Coming from organizational and management studies, the concept of institutional logics is very suited for studying the inner workings of organizations that are part of the strategic action fields (Fligstein and McAdam 2011) of which they have obtained a certain understanding. Thornton and Ocasio (1999) argue that institutional logics shape organizational behavior in three main ways: first, institutional logics provide the rules that determine the locus and legitimacy of power within organizations; second, institutional logics comprise a set of implicit rules that determine what issues are relevant to consider for actors within organizations; third, the values and beliefs that comprise institutional logics determine (influence) what (kinds of) problem-solving approaches are available to actors within an organization or industry to emergent problems, and what actions are appropriate (Thornton and Ocasio 1999). Institutional logics provide actors with the tools to engage with and understand the world, by specifically focusing on some aspects, and giving less attention to others. By adhering to a particular perspective on reality, actors develop legitimacy for and a sense of stability in undertaking actions within their work. Thus, institutional logics are crucial determinants of the evaluative criteria, operations and practices that are deemed acceptable and legitimate within a particular sector or organization (Geels 2020).

### Institutional work

One element of institutional theory, “Institutional Work”, is especially concerned with actors and their individual agency (Berg Johansen and Waldorff 2017). Institutional logics, determinant of standards and practices within a field or organization, require deliberate actions by individuals to disrupt them, or to create new institutions. Such actions are characterized by Lawrence et al. (2009) as ‘institutional work’, i.e., “the purposive action of individuals and organizations aimed at creating, maintaining and disrupting institutions” (Lawrence and Suddaby 2006, p. 215). Institutional work comprises three ideal types of action; maintenance, tinkering, and disruption. As actors are in continuous engagement with their relevant institutions, institutional logics are therefore better understood not as static, but as gradually (slowly) evolving under the influence of ongoing institutional work.

### Framing

Closely related to institutions and institutional logics, a frame is a “conceptual structure used in thinking” that individuals utilize to understand reality (Lakoff 2005). Taken-for-granted cognitive schemes are core aspects of institutions (Werner and Cornelissen 2014). They are self-evident and beyond evaluation unless they become a topic for deeper reflection. Reflexivity is the act of examining one’s assumptions, beliefs, and judgment system (Jamieson et al. 2023). Becoming aware of one’s frames of thinking and evaluation allows people to view a situation differently and become appreciative of other problem-solving approaches. According to Gray (2003), frames define specific issues, shape actions, and influence preferences (e.g., accepting certain responsibilities, openness to cooperation, and willingness to learn), in contrast to logics, which provide more overarching direction for organizational behavior. When frames are strategically applied, we speak of framing. Framing is “an active processual phenomenon that implies agency and contention at the level of reality construction” (Benford and Snow 2000). Werner and Cornelissen (2014) refer to different types of reframing based on similarity with or contrast to existing schemes and whether the change is minor or major. They suggest reframing can be partial (a change to the existing frame), provisional (temporary release of the dominant frame), and settled/unsettled (referring to the consensus or ambiguity surrounding the frame or competing frames) (Werner and Cornelissen 2014).

To understand the role of institutional dynamics, it is important to distinguish between institutional logics and frames. Although they are interrelated, ‘frames’ and ‘institutional logics’ serve distinct roles in shaping actor behavior and organizational responses. According to Benford and Snow (2000, p. 614) frames constitute a broad interpretive answer or definition to “what is going on” or “should be going on”. A frame thus applies to a specific situation and guides attention to specific aspects, while ignoring others. Frames and framing are closely related to cognitive and linguistic processes of association and schema building (Werner and Cornelissen 2014). This is how frames can influence institutional logics, which are more durable frameworks that define patterns of legitimate behavior, norms, and rules within a given institutional setting (Thornton and Ocasio 1999). In other words, frames guide the immediate perception and interpretation of a specific situation. They are rather narrow, flexible, subjective, and temporary. In contrast, institutional logics shape the fundamental assumptions and practices that underlie long-term institutional patterns of behavior. Discursive acts of framing and reframing may form the basis for institutional change (Benford and Snow 2000; Fligstein and McAdam 2011; Werner and Cornelissen 2014), and are therefore closely related to institutional work, since acts of reframing can be used strategically to reinforce or challenge dominant institutional logics.

### Conflicting frames and institutional logics

Building on the work of Franco-Torres et al. (2021), the present study is concerned with how organizations deal with conflicting institutional logics. While singular institutions and institutional logics are often dominant within sectoral organizations (Besharov and Smith 2014; Franco-Torres et al. 2021), it is also recognized that actors often are under pressure from several distinct institutional logics (Franco-Torres et al. 2021; Fuenfschilling and Truffer 2014). When these diverse logics are misaligned, actors and organizations may need to find ways to align them, or privilege a certain logic to guide their decision-making, particularly when facing sustainability challenges (Smink et al. 2014; Hestad et al. 2021). In co-creation projects where several actors develop solutions to shared problems, the confluence of several competing logics is inevitable, and balancing interests, costs and benefits can be challenging. Moreover, competing frames or (strategic) framing actions can contribute to the persistence or resolution of (perceived) conflicts in actor perspectives. For instance, a water management organization, being the focus of the present research, may be inclined to pursue actions that align with the perspective of local constituents, adhere to hydrological and technical standards proposed by technical experts, while also keeping in mind the cost-effectiveness and expedience of actions as required by higher management. Van der Wal et al. (2011) describe how public organizations regularly encounter conflicting value perspectives, and that such actors are often restricted in their decision-making due to institutionalized value systems and practices. In a 2013 study, Cook and colleagues found that even when participatory governance is emphasized, hierarchical practices often persist when there are multiple competing frames, while Geels (2020) highlights that socio-technical transitions are contested processes with competition between dominant logics and emerging templates. Tensions between dominant and emerging frames and institutional logics thus present a challenge to organizations looking to adapt to new societal demands in sustainability transitions. To overcome this challenge, organizations increasingly turn to design thinking to create space for creative solutions that satisfy the diverse needs of each actor.

### Design thinking as a strategy for managing conflicting institutional logics

In recent years, design thinking has gained attention within sustainability transitions as an approach by which to open-up the problem and solution spaces and generate innovative solutions to complex sustainability problems (Gaziulusoy and Ryan 2017; Irwin 2015; Maher et al. 2018; Öztekin and Gaziulusoy 2020; Simon 2023; Seiferth et al. 2024). The premise of iterative design cycles, phasing between divergent and convergent thinking [i.e., the double diamond (UK Design Council 2019)], shows promise for effectively dealing with competing institutional logics, as it allows for repeated exchanges and ideation surrounding a shared problem and incorporating each actor’s distinct perspective into the solution design. As such, a design-centered approach is suitable to the objective of the CARE project, namely: to (re)design spatial instruments for climate-adaptive water management in the Limburg region. The open-ended nature and broad framing of the task at hand are well served by the design principles that underpin design-thinking approaches, as stakeholders can exchange perspectives and knowledge and co-create a shared problem-framing, before collectively designing solutions in the form of (spatial) instruments. While design has received increasing attention in sustainability science and transitions literature (Simon 2023; Seiferth et al. 2024), its application in overcoming tensions between competing institutional logics remains underexplored. The current paper addresses this gap by investigating to what extent the design-based approach utilized in the CARE project contributed to the resolution of conflicts between institutional logics.

The theoretical concepts discussed in this section—ranging from institutional logics and institutional work to framing and (field) frames—provide a useful lens through which to investigate how and to what extent WL was able to navigate the complex institutional dynamics at play in CARE. Building on these theoretical insights, our study examines how the approach employed in the CARE project facilitated the co-creation of new institutional logics and the overcoming of conflicts between competing institutional logics and frames.

## Project context and description/case study overview

After the devastating 2021 flood event, Waterschap Limburg (WL) participated in the INTERREG EMFloodResilience project, established to promote climate resilience in response to these events. The flooding was caused by exceptionally heavy rain (a 48-h cloudburst) feeding the river systems in the region, leading to significant damage, including loss of life, in the river valleys (Hagenlocher et al. 2023). In response to the call for project proposals within the larger INTERREG EMFloodResilience research project, Waterschap Limburg proposed a 6-month investigation and (re)design of climate-adaptive registration instruments. Recognizing the increasing challenges posed by climate change (e.g., changing precipitation and weather patterns, increasing drought events), particularly after the 2021 flood event, Waterschap Limburg sought to develop tools that would enable a more adaptive and resilient approach to water management. This initiative was led by a senior manager responsible for the current registration instrument of the water authority, the ‘Legger’. This instrument, which is one of the oldest in Dutch water management, provides a systematic overview of assets such as channels, flood barriers, and other structures, as well as information about water levels, flow rates, land use, ownership, and relevant regulations. The objective of the project was to explore the potential benefits of developing a registration instrument that included climate adaptivity and (dynamic) information necessary for integrated water management.

Prior to this project, Waterschap Limburg had provided three employees with the opportunity to familiarize themselves with ‘design-thinking’ methods. The project initiator hoped that the application of a design-thinking approach would support out-of-the-box solutions and broaden the scope of the trajectory. To this end, a foresight and design agency was hired to develop and facilitate the project and its workshops. In addition to the primary aim of developing new instruments, one of the secondary aims of the CARE project was for the three employees to experience participating in a design-driven co-creation project. These factors contributed to the project’s aim of taking an integrated approach to climate-adaptive water management, using design methods to meaningfully engage stakeholders in co-creation, and innovating beyond a mere reconfiguration of the existing instruments used by the water authority. Recognizing the complexity associated with broadening the focus of the project, as well as the restrictions imposed by the project’s short timeline, the development of a complete tool and working prototype was determined to be unfeasible. Therefore, the project rather aimed to develop building blocks for an instrument.

Formally, the aims of the project were defined as examining suitable tools for climate-adaptive water management, in co-creation with stakeholders, and exploring innovative and creative methods such as are implied in design-based approaches. To these ends, the project featured three co-creation workshops aimed at designing building blocks for climate-adaptive water management instruments. The first workshop introduced the project, exploring actors’ values and improvement perspectives, gathering input from participants and developing a shared problem framing. The second workshop built on the insights gained from the first, using two design questions developed by the project team in response to the outcomes of the initial workshop: How can we make the Legger more climate adaptive and inclusive, and is it the right instrument for the project’s goals? How can we enhance the self-reliance and resilience of citizens with regard to climate adaptation and resilience? The latter design question was not part of the original scope of the project, but included in response to the perspectives and needs of citizens expressed during the first workshop. The third workshop used visioning exercises and customer journeys to investigate the practical application of the two conceptualized tools that resulted from the second workshop: *Klimaatonderlegger* (Climate underlayer), and *Iedereen waterbeheerder* (Everyone a water manager).

The results of the CARE project were concepts and building blocks for these two tools. First, the Klimaatonderlegger is a combination of spatial information layers (e.g., spatial planning, climate scenarios, ecological networks, regulatory information, etc.) to be used in deliberations between local governments and stakeholders. Second, the instrument Iedereen waterbeheerder is based on the same combination of information layers, but in this case presented in an accessible and understandable way to inform citizens of climate-related risks, as well as preventative and mitigation measures to independently implement in and around their homes.

The approach taken in the CARE project features several characteristics of design thinking. First, the project prioritized understanding the needs and perspectives of diverse stakeholders. Actors from the agricultural sector, entrepreneurs, civil servants from the province of Limburg and local municipalities, environmental NGOs, and citizens were invited to contribute to and co-create solutions within the workshops. Second, the project followed an iterative process where ideas and perspectives were continuously exchanged and refined within and between the three workshops. Third, the open-ended nature of the project allowed participants to come up with out-of-the-box solutions and ideas. This was evidenced by the resulting development of not one adjusted registration instrument (such as a revised version of the Legger), but rather two distinct instruments: one for regional collaboration among stakeholders, and another for empowering citizens’ resilience to droughts and floods through risk and damage mitigation. Fourth, collaborative co-creation, as employed in this case, is central to design thinking, and the diverse actors, through repeated participation in the design workshops, continuously contributed to the development of the instruments and to the project outcomes. Fifth, though limited due to the time constraints of the project, the strategy of early prototyping was employed through the use of ‘customer journeys’, which enabled participants of the workshops to consider user needs and develop concrete ideas about how the two instruments would be used in practice. Sixth, the CARE project was characterized by an integrated approach to water management. Beyond incremental adjustments, design thinking promotes holistic solutions to complex problems, as were developed during the CARE project. The integration of climate models, policy documents, spatial planning, and maintenance information into one database for the two tools reflects this integrated approach to the problem of climate-adaptive water management.

## Methodology

The data collection for this study was performed as part of a multiple-case collaborative research project into changing practices for sustainability transitions in the Dutch water sector (X). Within this project, the first author conducted embedded action research (McGinity and Salokangas 2014) for 4 years (2020–2024) at the CARE project’s lead organization, Waterschap Limburg (WL). As a researcher embedded in the organization, the first author obtained a deep familiarity with the culture of the organization (the written and unwritten rules, as well as the tensions, struggles, and animosities between people and departments) through conversations with people (planned and unplanned) and observations in meetings. At WL, the researcher was expected to support and facilitate thinking and innovations and transformations toward integrated practices, and observing and analyzing change processes and organizational dynamics (including the factors that constrained integrated thinking). In this capacity, the researcher was part of the ‘core team’ organizing and developing the CARE project throughout its runtime. The nature of the involvement of the (lead) researcher in the events of the CARE project is innovative and atypical in the study of institutional change. The embedded research approach enabled observation at close range, and to thereby yield detailed insights into the interactions in the workshops and core team meetings. While the embedded approach provides rich observations, it also induces potential challenges and biases due to the long-term and close interaction between the researcher and the organization studied. These concerns were mitigated through continued reflection and transparency with the host organization and broader research team.

Literature on co-production often engages with the concept of boundary work or boundary spanning (Bednarek et al. 2018; Velter et al. 2020; Zeigermann and Ettelt 2023; Zietsma and Lawrence 2010); we focus on institutional logics and frames to explicitly investigate systemic factors that shape organizational behavior and change to retain a focus on internal change processes. This perspective enables us to focus on the varied nature of institutional norms and structures within organizations, and offers insight into the role of competing logics and frames in co-production processes. Drawing on concepts from institutional theory heuristically, we interpret the emerging tensions within the water authority as manifestations of conflicting or competing institutional logics and frames. The interpretive nature of rules and frames calls for an interpretive type of analysis which is often absent in numbers-oriented research (Engström et al. 2023; Hirsch-Hadorn et al. 2008).

To enable critical reflection on the CARE project, the design approach employed, and the effects on competing frames and institutional logics, several ethnographic methods were used. First, as participant observer (taking part in each workshop as a participant while recording observations through concurrent and post hoc note taking), insights were obtained from the three design workshops to understand how WL employees as well as external stakeholders engaged with and negotiated competing frames and institutional logics throughout the project. These longitudinal observations were collected in detailed contemporaneous and post hoc notes which offered detailed accounts of participants’ attitudes and interactions (as expressed via words and physical acts), which helped to obtain a micro-level view into the dynamics between stakeholders (revealing differences in perspectives, assumptions, and valuation). Second, field notes from core team meetings, informal interactions, and project documentation offered a comprehensive overview of the CARE project’s evolution, and considerations and decisions made by the core team. These documents provided insight into the project’s context, background, iterative development, and final outcomes. Third, semi-structured interviews were conducted with three key participants, selected using the criteria that they were employed at WL and remained attached to the project and the core team throughout its runtime. Each of the participants was interviewed before and after each workshop session using open and evaluative questions to guide the interviews. Methodologically, it was an explicit choice to have multiple interviews with 3 participants, for ascertaining the (changing) attitudes and experiences of the interviewees throughout the lifetime of the project, the learning process about the topics discussed, and salient outcomes of the design-based process. This resulted in a total of 12 interviews, four per participant, between 30 and 60 min per interview. An additional goal of the interviews was to obtain clear insights into the perceived value and contribution of the design-based approaches for navigating competing institutional logics. The primary data from these three sources was analyzed using an interpretative approach, based on the theoretical concepts and frameworks elaborated in “Theoretical background”. Together, the analysis of these data sources enabled a comprehensive exploration of how and to what extent design thinking facilitated participants in the CARE project in managing the complex institutional dynamics at play.

The workshops involved participants from diverse departments within the organization, reflecting a broad spectrum of perspectives relevant to the design-based project. While attendance lists were not maintained for individual sessions, the number of participants invited and those who attended each session were recorded, as shown in Table 1.

**Table 1:** Workshop participation overview

Workshop session	Participants invited	Participants attended	Dropout (from previous session)	Dropout (%)
Workshop 1	40	34	N/A	N/A
Workshop 2	40	32	2	5.9
Workshop 3	40	31	1	3.1

Attendance remained relatively consistent across the three workshops, with minimal dropout between sessions. The dropout rate was 5.9% after Workshop 1 and 3.1% after Workshop 2, with most dropouts resulting from scheduling conflicts. These numbers suggest high participant commitment to the workshop series. The participants included WL employees, representatives from other local governments, NGOs, local entrepreneurs, and citizens, reflecting a mix of roles and expertise.

## Navigating diverse institutional logics in the CARE project

Following the approval of the initial proposal by the funding agency (INTERREG), WL’s internal project team prepared a request for proposals (RFP) to distribute among companies specializing in design thinking projects. They sought facilitators for the upcoming initiative and received two proposals in response to the RFP: one detailing a 2-week ‘design-sprint’, and another detailing a longer, more inclusive, and iterative process. Although WL commonly operates in accordance with a logic of efficiency, minimizing costs and demands in terms of time and staff, the project team decided to deviate from this norm, upon suggestion from one of the core team members to ‘try new things’. The unique status of the project, with external funding and with research as its primary objective, gave this person the impression that this was an opportunity to experiment in the selection process. They requested a colleague unaffiliated with the initiative to redact price indications from the two proposals, to enable selection based on quality, rather than cost estimates. During an initial round of appraisal, each project team member selected their preferred proposal. Unaware of the costs, the team unanimously selected the same proposal, which featured more stakeholder interaction, iterative cycles of development, and displayed more knowledge and understanding of the water sector, and the project’s goals. Following this selection, the unredacted proposals were shown in the meeting, revealing that their choice was also the least expensive. While this validated the prior selection to the project team, individual members expressed that a higher cost would not have discouraged them from granting the project to the chosen party. The selection process highlights a clear departure from the dominant efficiency logic, which strongly favor cost- and time-effectiveness and therefore would align more with the design sprint proposal. The choice in favor of a longer and more inclusive process reveals a willingness among the members of the project team to deviate from dominant institutional logics.

After the project was granted to the selected party, a Belgian foresight and design company, Pantopicon, the initiative entered a phase of developing a plan of approach for the project. Pantopicon worked closely with the internal project team to clarify objectives, align expectations, and develop a course of action for the implementation of the project. During meetings with the project team, discussions led to a broadening of the original project scope to developing concepts and building-blocks for tools to be used for integrated and climate-adaptive water management, rather than focusing solely on the re-design of the incumbent instrument, the Legger. This expansion reflects a strategic shift toward addressing broader environmental and societal needs within the project. One interviewee later stated: “Taking a broader perspective is often seen as contradictory to carrying out core tasks in a focused and efficient way.” What is interesting to note, however, is the difference between layers of the organization: between management and operational staff, in the way they think about their work and the role of WL. “People at our [operational] level are happy to try new things and take initiative,” said one interviewee. They are more likely to do this than top management. As another noted: “The board and management are apprehensive to innovations, and prefer top-down control. When it comes to new practices or thinking ‘out-of-the-box’, they leave the initiative to us [operational staff], to avoid accountability if it ends up not working out.” The broadening of the project scope reflects a competing institutional logic, prioritizing innovation and societal value that challenges the dominant logic of the organization, prioritizing core tasks and efficiency. This highlights the (limits to) agency of employees by showing their willingness to take opportunities to challenge the dominant logic and engage in institutional work, even with little perceived organizational support.

Following the initial meetings, Pantopicon developed a three-step approach to designing instruments for climate-adaptive and inclusive water management in Limburg, featuring three co-design workshops with relevant stakeholders. While there was a priori agreement on the content and format of each session, the project team opted for an iterative approach with continuous reflection and re-adjustment that would allow the outcomes from each workshop to inform the format and focus of the subsequent sessions. This iterative approach not only supported stakeholder buy-in, but also ensured that the project would be responsive and attentive to evolving needs and objectives from participants.

### Workshop 1

During the first co-design session, stakeholders were introduced to the concept of integrated water management and the diverse social, ecological, and governance factors involved in climate adaptation. Using their experience in transition studies and water management, two experts (i.e., a hydrological expert from WL, and a water policy expert hired by Pantopicon) presented analyses of relevant developments, partnerships, and current legislation on the European, national, and regional scale. The introduction of integrated water management represents a competing institutional logic, favoring more holistic and participatory approaches, that challenges the professional and efficiency logics dominant at WL. Surprisingly, there were no objections to the new logic from attending WL employees, though they acknowledged that following this logic would imply substantial changes to current practices. One WL employee noted that “the role of WL in the region may require some revision,” while another rhetorically asked, “Is [WL] good enough the way it currently is?”, showing early recognition of the required shift. The workshop’s initial objective was to probe participants and develop a shared understanding of integrated and climate-adaptive water management. In the first interactive segment of the workshop, stakeholders were randomly divided into smaller groups and tasked with identifying current challenges facing the water authority and society at large in relation to climate-adaptive water management. Each group's discussions were recorded on pieces of paper and subsequently shared and discussed in a plenary session with all workshop participants. The challenges identified ranged from climate change to legal barriers and political tensions. Following the central discussion, stakeholders engaged in a second round of group discussions, focusing on potential future challenges that could influence water management. The facilitators provided prompt-cards featuring disruptive future developments to stimulate conversations. The insights gained from this initial workshop provided a basis for identifying key priorities and next steps in the project, but also underscore the emerging tension between integrated water management practices and the entrenched logics within WL.

This first workshop enabled stakeholders to introduce new and diverse perspectives that challenged established norms within WL, and thereby participants set the agenda for the remainder of the project. For instance, participants highlighted the need for community engagement and effective provision of relevant information to citizens. The shift toward inclusivity not only aligns with the project’s goals, but also with broader societal trends toward participatory governance. Including new perspectives and objectives introduced by actors after the start of the project is partially at odds with the dominant logics of control and efficiency, and therefore produces tension with incumbent institutional norms. The first workshop served as a site for initial negotiations regarding entrenched frames and institutional logics. In its objective to redefine the role of WL, as well as one of its primary instruments, the project represents an attempt to shift the dominant institutional logic at WL toward emerging logics that support more adaptive and inclusive water management.

### Workshop 2

Informed by the discussions and contributions of participants during the first workshop, Pantopicon summarized the perspectives shared and issues raised and organized them along two main themes: redesigning the Legger for co-governance, and developing a similar instrument to be used by other stakeholders. Based on these two themes, the core team developed two questions to guide the second workshop: 1. How can we make the Legger more climate adaptive and effective? and the sub-question Is the Legger the correct instrument for this purpose? 2. How can we enhance the self-reliance and resilience of societal stakeholders (especially residents and entrepreneurs) concerning climate adaptation? This second question, while outside the original scope of the project, was included to respond to the demands and perspectives expressed by citizens during the initial workshop. The core team’s decision to address self-reliance and resilience speaks to a logic of social responsibility and adaptive governance. The second workshop tasked participants with answering these questions, split into four groups, two dedicated to each question.

The groups concerned with question 1 engaged in clear institutional work. The question involved ‘tinkering’ with the existing instrument and its use in practice to develop new practices and perspectives to support climate adaptation. This was exemplified in a discussion between a WL employee and a local government representative, who stated that “[…] the legger should contain information about the entire sponge [i.e., the regional water system]”. They discussed how such an instrument could contribute to climate-adaptive spatial planning through stimulating co-governance between the water authority and local governments. This represents an adjustment of the current instrument, which merely reports on (the state of, and laws applying to) distinct natural and artificial objects within the water system. The current instrument is a material feature of the dominant logics (efficiency, control), while the adjusted ‘Legger’ represents a new logic (integrated water management). Another WL employee discussed with a citizen how she was skeptical about the feasibility of using a single instrument to “steer” all aspects of regional water management. In her view, this would cause the water authority to overstep the boundaries of its mandate and would require adjusting national legislation regarding the tasks and jurisdiction of water authorities in the Netherlands. Several other participants highlighted the issue of control and jurisdiction as potential barriers for the intended instrument, reflecting institutional and legal constraints to the proposed innovation. Here, the entrenched logic is at odds with creative innovation of the existing instrument, and with the idea that WL could adopt a new (stronger) role in regional spatial governance.

These discussions reveal a tension between the current institutional and governance arrangements, and those seen as possible and desirable by the participants in the workshop. As highlighted previously, the water authority is primarily concerned with its core tasks, which are conservatively defined and operationalized. In practice, this conservative approach to the operations of WL manifests through the risk-averse behavior of employees, and their apprehensions about organizational support mentioned previously. Developing an instrument for climate-adaptive water management, aimed at co-governance of spatial planning issues together with local governments, therefore is at odds with WL’s current characterization of its tasks and its role. This is a manifestation of competing institutional logics, and of the design process pushing the boundaries of logics that are dominant at WL. Interestingly, challenges to dominant logics were mounted by external stakeholders as well as organizational members, for example highlighting inclusion by addressing ‘hard-to-reach stakeholders’. Other discussions in this workshop circumvented the issue of radical change, and engaged in incremental change, making marginal adjustments to, for example, include measures of drought in the current instrument. This instance of institutional ‘tinkering’ reflects an effort to reconcile the novel solutions with existing norms of practice and highlights the agency of individuals within an organization. The difference between those proposing radical changes and those ‘tinkering’ with existing practices highlights the diversity in institutional entrepreneurism among employees.

In the groups that were assigned to question 2, it became evident that citizens were primarily concerned with risk mitigation, safety, and receiving support for mitigation and adaptation efforts. This led to the development of the concept for a second tool; “iedereen waterbeheerder” (everyone a water manager). The participating citizens and entrepreneurs (i.e., agrarians, store owners, and representatives from a regional development agency) were enthusiastic about the idea of increasing their own knowledge and capacity for dealing with water-related risks and developments. The idea emerged for ‘water coaches’, as expert intermediaries to facilitate the adoption of climate-adaptive and resilient measures at home, and this concept was supported by the participating WL employees. Moreover, entrepreneurs expressed a need for clarity on regulations and support for economic viability while contributing to climate adaptation.

The discussions in these groups reveal competing institutional logics: community empowerment and decentralized water management on one side, and WL’s traditional logic of centralized control on the other. While the dominant logic positions WL as the primary actor in water management, protecting and providing for other actors, the “iedereen waterbeheerder” concept reflects a shift toward participatory governance. The enthusiasm expressed for this concept fits to a broader movement toward participatory governance (Carvalho et al. 2019), which contrasts with the logic of centralized and control-oriented governance as dominant within WL. Entrepreneurs’ concerns about regulations and economic viability further indicate a need for institutional adaptability. For example, in one group discussion, entrepreneurs and WL employees proposed incorporating water retention and infiltration measures in the design of new commercial property developments, provided there is clear support in terms of regulation, technical knowledge and economic feasibility. This represents a significant departure from the dominant view of water management tasks within WL, which positions the organization as the dominant actor in water management. The alternative frame, where citizens and external actors are substantially more involved in responding to and mitigating risks, is selectively activated for the purpose of the design workshop, though it is at odds with the daily operations of WL. The reframing, in this instance is provisional, as it is meant to create temporary space for the innovation, but without committing to an enduring shift in perspective.

Overall, the interactions and outcomes of the second workshop reveal tensions between existing institutional logics and practices, and emerging perspectives on climate adaptation. The participants’ suggestions show opportunities as well as challenges in aligning institutional standards with novel climate-adaptive strategies. The design workshop appeared to provide a ‘protected space’ (Geels 2014; Smith and Raven 2012), where novel perspectives can be introduced and established practices can be questioned. The absence of higher management figures, the collective discussions, and the diverse mix of participants likely contributed to this environment. The application of this project format may therefore have supported participants in navigating and finding ways to overcome tensions.

### Workshop 3

Following the second workshop, the core team reflected on the outcomes of the previous sessions and designed the third workshop around two distinct concepts: 1. an integrative spatial tool (the klimaatonderlegger, or climate underlay) aimed at facilitating co-governance between the water authority and local governments, and 2. “iedereen waterbeheerder” (everyone a water manager) aimed at increasing the self-reliance and resilience of residents and shop owners in flood-prone areas. Both instruments would rely on a comprehensive integration of available maps, climate models, policy information and other sources, but the instruments would differ in how they present this information to users. Specifically, co-governance would require an instrument that would provide users with a comprehensive overview of legislative, regulatory, and spatial–physical information such as geomorphological maps and climate models, and information on planned and current policy, to support discussions on policy alternatives. Meanwhile, the instrument for citizens would simplify the same data, to make it usable for ordinary citizens who may lack the expertise to interpret complex data. Furthermore, the instrument would link this information to practical mitigation and adaptation measures for citizens and entrepreneurs. To further investigate the needs of each user group, the third workshop was designed around the use of ‘customer journeys’, to co-design the instruments together with potential users.

In the third and final workshop, participants chose to develop customer journeys for either of the two instruments, based on the relevance to their individual situation and personal interest. A customer journey exercise is an approach used in design to map the experiences and interactions that a customer or user has with a product. Pantopicon provided formats to guide the discussions, helping participants to identify ‘touchpoints’ (moments or situations when users come into contact with the instrument), and key interactions (how users engage with the instrument). Even though the instruments had not yet been developed into prototypes, this exercise enabled participants to envision themselves using the instruments in practice, thereby iteratively identifying important elements and mechanisms of interaction. The touchpoints identified ranged from a digital landing page, interactive maps, videos, 3D simulations, to local knowledge brokers, water coaches, and community ambassadors. The group discussions around “iedereen waterbeheerder” centered around two main themes: a desire from stakeholders for 1. transparency and insight in public (spatial) planning policies, and 2. the provision of comprehensive practical knowledge for risk mitigation and climate adaptation. In addressing these two objectives, citizens and entrepreneurs are empowered to play an active role in adapting to and mitigating the effects of climate change on the water system.

The above interactions reveal tension between incumbent frames and practices at the water authority, and the ideas developed during the design workshops. The approach to citizen empowerment that emerged from the workshops in the CARE project is different in several minor, but important ways from the currently dominant practices used by WL to engage citizens. While the water authority aims to involve local citizens in plans that impact their direct environment, this is often limited to incidental community events where citizens can voice concerns, ask questions or object to developments. The approach developed in the CARE project reveals an interest from citizens in being more directly involved in taking mitigation and adaptation measures, provided that they are supported and informed by the relevant parties. A member of the core team reflected on this tension, stating that: “[…] employees are often scared to involve citizens more than necessary, since it could lead to additional burdens of legitimation, delays, and higher costs or workload.” Moreover, he highlighted that “[…] citizens suffer participation fatigue, especially when they feel as though their involvement is performative.” Another member of the core team mentioned that “[…] project leaders often prefer simply executing a process without too much citizen engagement, so that there are no additional objections or considerations that could threaten the planned process.” Again, these impressions produce a nuanced perspective on the institutional logics involved in WL operations. On the one hand, WL aims to engage with citizens wherever possible to generate support for its projects, and gain legitimacy. On the other hand, it appears that the citizens involved in the design workshops of the CARE project suggested that they themselves could play a more direct role in the climate-adaptive management of the water system, by being better informed, taking measures on their own properties, and being prepared for future climate scenarios. Nevertheless, the aforementioned objections to the involvement of citizens remain present in the broader organization, thus reflecting partial and provisional reframing, and potentially hindering the uptake or further development of the project’s outcomes.

In the groups concerned with developing the ‘klimaatonderlegger’, participants discussed the challenges associated with developing a spatial decision support instrument. A key technical challenge identified was the combination and presentation of complex information from several sources in a comprehensive and dynamic manner. For instance, climate models are future-oriented projections, while zoning and spatial policy plans are concrete and binding interventions in the environment. Combining these diverse layers of information and presenting an overview that enables informed deliberations between governments presents significant technical challenges. From a governance perspective, the challenge lies in obtaining commitment from local governments and other stakeholders to use the proposed instrument to align policy with information about the water system, climate change risks and environmental protection objectives. A participant from a local citizen council expressed that “If it turns out to be inconvenient for policymakers to adjust their plans to all these considerations, won’t they just ignore it and go ahead as usual?”, highlighting that, beyond the design of the tool itself, aligning policies also requires addressing the political realities of co-governance. This prompted another person in the group to propose that “The use of this instrument should be formally established in legal and governance arrangements.” This proposal was seconded by a core team member in an interview, who reflected on the potential implications of the ‘klimaatonderlegger’ for the role of the water authority in the region: “If we would have such an instrument, we would be able to inform and perhaps influence the spatial policies of other governments. Right now, even though their policies have direct impacts on the water system, and by extension, our work [at WL], we are not allowed to give our opinion, as it is outside of our jurisdiction. A lot of our colleagues are afraid of ‘overstepping WL’s authority’.”

Interestingly, of the two groups tasked with developing the klimaatonderlegger, one group revealed they had substantial concerns about the issue of jurisdiction, and saw it as a barrier, while the other continued the design process under the assumption that this issue would be overcome, through agreements between authorities, or new governance arrangements and legislation. This reveals further tensions within the water authority, as one group adheres strongly to a logic of bureaucracy, control and compliance, while the other is aligned with a logic that favors innovation and adaptability. Reflecting on this discussion, a core team member noted: “I think that people feel uncomfortable with such an integrated perspective, because it implies a new role for the water authority. When colleagues then indicate that taking initiative would require changes to legislation and governance arrangements, I say: In that case let’s adjust the governance arrangements!”, showing a willingness to engage in disruptive institutional work. This perspective is a sharp contrast to the one commonly adopted within WL—top-down, bureaucratic operations, in compliance with existing arrangements—but it reveals that there are some within the organization who dare to look beyond the current status quo to find innovative solutions, even if these could require the redesign of governance arrangements.

### Post-phase

Following the workshops the concepts for the two instruments were elaborated and reported by Pantopicon, in collaboration with the core team. The workshop proceedings and results were bundled in a project report for internal dissemination, as well as summative presentation slides. Results were presented by the project lead to the management team, as well as the daily board, and were initially met with interest and enthusiasm. A limited follow-up project employed three trainees to develop a small-scale prototype of the spatial decision tool ‘klimaatonderlegger,’ and their working prototype was presented to a small group of interested colleagues. Although the report and prototype did not prompt further investment from the management or board, they provided input for a new collaboration project that had been started together with the provincial government. Initiated by the province, this project aims at developing shared spatial planning information to support collaboration among governments on soil- and water-related policies. While the project draws on similar arguments and insights as CARE, it is unclear to what extent the new project utilizes the knowledge gained from both the original project and its follow-up.

## Discussion

The following section will draw connections between the findings presented in the previous section and discuss their broader implications for research and practice. We critically examine how our findings contribute to understanding (competing or conflicting) institutional logics, frames, design thinking and their application within the water sector. We compare our findings to existing literature, consider the practical implications of our findings for both theory and practice, and assess the strengths and limitations of the methods employed.

Several institutional logics can be of influence at the same time, and even within organizations, logics may not be applied consistently. The level of ascription to a (set of) logic(s) may also differ between managerial layers, teams, or even individual members of an organization. We found instances of certain WL employees committed to the dominant logics (i.e., efficiency logic, bureaucratic logic), while others appeared to align with logics of community empowerment and water sensitivity. Even within projects, several logics are often at play simultaneously, for example community empowerment in phases of legitimation and validation with citizens, though market and efficiency logics are applied during implementation. Diverse logics can thus co-exist within a field or organization, while being selectively activated or competing for dominance if these logics are not mutually reinforcing or compatible. This is in line with previous studies (Franco-Torres et al. 2021; Greenwood et al. 2010; Besharov and Smith 2014) that highlight how organizations are often under the influence of competing institutional logics that are sometimes incompatible. Our findings thus support the notion that institutional logics are fluid and contested, varying between and within organizations to influence decision-making and efforts at problem-solving. Specifically, the WL case shows that efficiency logic, of performing existing tasks in a (cost-) efficient manner, is dominant within the organization, while other logics play a peripheral role. The persistence of (tensions between) multiple institutional logics may not be easily resolved and could indicate an institutional complexity where actors ‘dynamically balance’ multiple competing logics (Smets et al. 2015). Though this allows individuals flexibility in following institutional logics, it also highlights that existing logics may not be readily transformed but are often selectively applied by workers (Besharov and Smith 2014).

While this study has primarily examined competing and conflicting institutional logics, we recognize that other relationships between institutional logics are conceivable. Besharov and Smith (2014), for instance, indicate the potential for logics to coexist within organizations, mutually reinforcing each other, or remaining plural. However, much of the literature on (multiple) institutional logics, including Besharov and Smith’s (2014) article, emphasizes competition and conflict in understanding the interplay between diverse logics (Smink et al. 2014; Greenwood et al. 2010; Hestad et al. 2021; Franco-Torres et al. 2021). This emphasis is reflected in the present study, supported by the salience of competition and conflict observed throughout the 4-year embedded research project. Future research may investigate settings where logics coexist or mutually reinforce one another to develop a more nuanced understanding of the interactions between institutional logics.

Reflecting on existing discussions of design thinking (Gaziulusoy and Ryan 2017; Maher et al. 2018; Öztekin and Gaziulusoy 2020), the process employed in the CARE project does not entirely adhere to design thinking as a process involving prototyping and testing. Specifically, given the broad objectives and complexity of the intended instruments, there was no possibility to engage stakeholders and end users in prototyping, as prototypes could not be developed quickly enough. Furthermore, design thinking is traditionally employed for issues with a more limited scope. The problem framing that was the starting point of the first workshop was broad and rather loosely defined, while common DT processes start with participants collectively defining a clear problem for which to design a solution. Another unique feature of the CARE project is the social and governance implications of the proposed solutions that emerged from co-creation. While the instruments can potentially aid policymakers as well as citizens in contributing to climate adaptation and mitigation, they implicitly demand that these actors adopt new and more proactive positions toward water management. Such innovations in social and governance arrangements are difficult to tackle using typical design-thinking methods. Therefore, while the project is grounded in design thinking, it differs from traditional design-thinking projects in terms of scope and the type of questions that were addressed.

These differences raise important implications for the application of design-thinking methodologies for complex multifaceted issues. Specifically, this suggests that a more flexible interpretation of design thinking may be needed to accommodate more unconventional applications of the methodology. The CARE project’s deviation from traditional DT approaches may call into question the generalizability of our findings regarding the effect of design-thinking methods on organizational change. We argue, however, that the core principles of design thinking (i.e., co-creation, iteration, divergent and convergent thinking) were still applied effectively, supporting the relevance of our findings to the study of design thinking.

The design approach employed allowed for unprecedented levels of adjustment and realignment, broadening the scope, and incorporating new perspectives and objectives as the project unfolded. Instead of focusing on a predefined target for the water authority, the project revolved around the needs, ambitions, and capabilities of the stakeholders present. This was a completely novel approach for the water authority, and a new experience for the participating employees. As a particularly effective element for reframing and challenging institutional logics, the customer journeys allowed participants to imagine the experience of other actors in interaction with the proposed instruments. The application of this method led to valuable insights for the involved actors, while also increasing empathy and understanding for the positions of other actors.

The current paper underlines the relevance of institutional theory for examining complex transitions processes in the water sector, a notion supported by the work of Fuenfschilling and Truffer (2014) and Franco-Torres et al. (2021). Fuenfschilling and Truffer (2014) identify three distinct institutional logics that affect actors in the water sector; a water market logic, hydraulic logic, and water sensitive logic. The current study, however, deals with a uniquely positioned actor that differs in important ways from the urban water sector studied in previous institutional research (Franco-Torres et al. 2021; Fuenfschilling and Truffer 2014). While existing research of this kind tends to cover water and sanitation services in cities, WL, as the focus of our investigation, does not provide these services but is responsible for water safety (flood-protection), water security (water quantity) and water quality (rivers, channels and tributaries, groundwater). Still, the identification of competing and sometimes conflicting institutional logics provides a useful lens through which to understand the change and maintenance of dominant institutional logics. The use of the concept of framing helped to ascertain the various interpretations of institutional logics by organizational members in relation to specific topics of discussion. Framing is an example of institutional work, which may result in new institutional arrangements. An example of this can be seen in the diverse interpretations of the assignment in Workshop 3. Two employees of WL had rather opposing views on the potential development of the ‘klimaatonderlegger,’ as it would require the adjustment of governance arrangements. Whereas one colleague considered this an insurmountable barrier, thus maintaining the existing institutional order, another expressed the view that, if needed, governance arrangements could be adjusted. This example shows that individuals within an organization may differ in their capacity or willingness to adopt (radically) new frames, and thereby highlights the contingent impact of both dominant logics, as well as efforts at institutional work. Moreover, our findings show that institutional work efforts within the project were diverse, with different participants ‘maintaining’ (through adherence to existing institutions and practices), ‘tinkering’ (by making marginal adjustments), and ‘disrupting’ (by proposing new institutional arrangements) the entrenched logics. These diverse responses during the project reflect what Geels (2020) calls discursive and institutional struggles around competing logics in transitions processes.

In the instances of reframing observed in our case, reframing was partial, provisional and unsettled. Partial reframing occurred when the project team framed CARE as an opportunity for trying new practices, and thereby temporarily released the institutional logic of efficiency to prioritize quality over cost in the selection of the facilitating party. The reframing is provisional, in that the new frame is applied singularly, and it remains uncertain whether this new approach will be applied in future selection procedures. Such temporary adjustment is indicative of selective activation and use of alternative frames, as is common and preferred to more radical or enduring adjustment of institutional logics within WL. The application of new frames can be viewed as instances of institutional work, as the situational adoption of a frame can challenge the overarching norms and practices that commonly guide organizational behavior. This is in line with a publication by Cook et al. (2013), which highlights that ‘normal’ top-down governance often persists in environmental management despite incidental efforts toward increasing participation and inclusion, while emphasizing the agency of individual employees to challenge dominant practices. The partial and provisional nature of the reframing observed in our case underscores the importance of the organizational context in understanding the impact of incremental or temporary changes in framing on persistent institutional logics and highlights the difference in scope and timescale between institutional logics and frames.

Several interview responses highlight that the CARE project’s format, as well as its outcomes, are perceived to be at odds with the current institutional standards of WL. Others, however, indicated that the current guiding principles of the organization (efficiency and professional) can be reinterpreted to align with and include new practices. This represents another instance of reframing, as core operations and guiding principles of the dominant logic are reinterpreted. Still, several interviewees stated that the majority of management and directorial staff at WL adhere to a more conservative interpretation. Though internal policy documents reflect ambitions for inclusive, participatory, and innovative projects, it appears that the support for these initiatives is often lacking in practice. This inconsistency highlights a significant gap between the organization’s stated goals and the organizational behavior that follows the dominant institutional logics in practice. When it comes to daily operations, this gap is often resolved through selective application of (strategic) frames. When it is convenient, and when the perceived risk of failure is low, innovations will be embraced and outwardly celebrated, while in other cases, they may be rejected for being inefficient or unneeded.

The discovery of a new frame related to the concept of *iedereen waterbeheerder* was an unplanned and unanticipated result. Whether it will be adopted by top management remains to be seen, highlighting another example of provisional reframing, but even when it is rejected for use by the present management board it is present in the mindset of several employees and represents a potential seed for (institutional) change. Those employees could potentially take on a role as institutional entrepreneurs driving efforts at institutional work. The new frame does not only concern attitudes toward collaboration, but also a reimagining of practices specifically related to the use of an existing instrument by and for the benefit of external stakeholders. More collaborative action with policymakers and other regional water authorities is likely to happen, given the interdependencies which became clearer and are better understood now. The present case provides empirical support for the idea that prospective thinking and a format of open deliberations in a learning-oriented process promote reflexivity in practice (Lawrence et al. 2009).

Reflecting on the embedded research approach, we find that gaining familiarity with the organization, and people in it, was clearly beneficial to the research. Academic research often fails to recognize the importance of practical activities and lived experiences of individuals within (policy) organizations (Ahrens and Khalifa 2013; Buchanan and Badham 1999; McIntosh and Wright 2019). However, collaborative action research can incorporate more interpretative approaches to research and learning (Croeser et al. 2024) and provide unique insights into micro-level interactions (Goldschmeding et al. 2024a). Being embedded within WL facilitated a continuous exchange between the academic and practical aspects of the research project. This ongoing dialogue was crucial for realigning expectations and action strategies, especially as the project evolved in ways that deviated from initial expectations set by the organization. This exchange allowed for a more adaptive and responsive research process (e.g., applying new selection criteria, broadening the project scope), which was essential in navigating the unpredictable challenges that arose.

While supportive of in-depth observation, the embedded nature of the researcher also introduced unique challenges related to his potential impact on outcomes. In a dual role, as advisor to the water authority while simultaneously conducting observational research, the first author had to balance between influencing and observing the process. This dual role poses the risk of influencing project outcomes, and of affecting the objectivity of observations and research findings. Therefore, the influence of the (presence of the) researcher must be considered when interpreting the findings, as it may have led to biases in participant responses. However, the dual role enabled the researcher to build rapport with participants, which may have helped to mitigate potential biases. Throughout the workshops and interviews, participants indicated that they felt comfortable sharing their concerns and opinions. Their openness was crucial in fostering a safe environment for dialogue and likely contributed to richer data collection. The first author’s active participation, including posing questions and facilitating discussion was viewed as a means to enhance engagement rather than introducing bias, though it potentially directed attention toward or away from specific issues.

To address these concerns, the researcher maintained transparency with project participants and stakeholders regarding research aims and methods and engaged in regular reflective sessions with the project team to ensure that his influence remained minimal. Potential biases induced by the direct involvement of the first author were mitigated through continuous discussion among the authoring team. Furthermore, it is important to note that the researcher was primarily concerned with organizational and institutional change dynamics, and the impact of the chosen design-based approach, rather than specific outcomes of the project.

As noted by Cook et al. (2013), competing frames regarding participation in governance can undermine the transformative potential of participatory initiatives. Thus, to foster enduring transformation of institutional logics and frames, (public) organizations should assess the alignment between their stated objectives and organizational norms and practices, as well as emerging conditions of the (social) environment. Such reflexive behavior can be initiated by employees at all levels but may be substantially more effective when performed in cooperation with those at the management level. Still, our findings show that management plays a crucial role in supporting and enabling the adoption of innovations and new ways of working, whereas a lack of support represents a significant barrier to employees’ institutional work efforts. To facilitate such institutional work behavior, conditions of pluralism, reflexivity and iterative practice can contribute to an environment conducive to challenging or transforming entrenched institutional logics and practices (Goldschmeding et al. 2024b). In the protected space of the CARE workshops, these three features were present as the process was iterative, engaged critically with taken-for-granted positions and drew in perspectives from a diverse set of actors. This likely supported participants in critically engaging with others’ perspectives, and challenging dominant, and working toward novel, emergent, institutional logics.

The willingness of participants to engage critically with existing, and develop new institutional logics has the potential for spillover effects beyond the project’s boundaries. However, these spillover effects warrant a more systemic investigation to draw robust conclusions about the potential for such spillover effects to emerge from similar projects in other settings. There is indeed some observational evidence for spillover effects among a subset of the participants employed by WL. It is likely that participants employed at WL were encouraged through the CARE project to adapt a more critical mindset in their everyday work. This aligns with literature on innovation culture (Tian et al. 2018), which emphasizes the importance of creating environments that support risk-taking and collective problem-solving to overcome challenges by fostering psychological safety and reducing power distance (Zhang et al. 2023). However, our observations at WL have revealed a lack of psychological safety and support for innovation. Such conditions may exacerbate existing challenges for novel practices and perspectives to break through beyond the protected environment of the CARE project (Smith and Raven 2012).

## Conclusion

The present paper has provided insight into how employees in a public organization in the water sector engaged with tensions between competing and sometimes conflicting institutional logics and frames in a design-based project. The project studied reveals significant tensions and potential shifts in institutional logics within the organization and the wider field, as well as diverse instances of institutional work. Through a process of design thinking and intensive stakeholder engagement new perspectives and opportunities for reframing emerged that challenged pervasive institutional logics within WL. The findings illustrate how iterative, participatory processes can lead to provisional reframing of (incumbent) institutional norms, while also showing that such changes are not easily integrated into the organization’s broader practices.

In doing so, this study broadens our understanding of institutional dynamics in public organizations, particularly in relation to complex transitions challenges such as climate change adaptation in the water sector. The findings highlight the potential of design-thinking approaches as tools not just for collective integrated problem-solving, but also for (temporarily) releasing and challenging dominant institutional norms and frames. We show that although design thinking is not typically used for the co-creation of alternative frames and logics, its methods and processes can help actors to engage in institutional work and reframing. The focus on tools brought into focus aspects of actionability. For academics focused on theory, the importance of this for practice is easily overlooked.

Institutional logics of organizations are often not formalized, they are multiple and subject to interpretation. Agency is enacted within structural contexts of institutional logics that structure the agency of actors, through dispositions and rules. Agency against structure consists of reinterpretation of the structures and the replacement, adjustment, and blending of rules and creation of new rules—engaging in institutional work. As such, the strategic and selective application of (novel) frames can be used to challenge or reinforce established institutional norms and practices. We find the institutional perspective useful for understanding prevailing organizational practices and responses to pressures for change.

Theoretically, our research contributes to organizational and transitions literature by empirically demonstrating how participants challenged institutional logics in practice, and highlights the relevance of institutional theory for studying organizational change dynamics. Practically, the study underscores the potential for design-based practices to enable institutional work, while identifying a need for continued efforts to integrate new approaches in organizational standards and practices.

Although this study provides some broader insights into institutional change, it is based on a case study of a single project, which limits the generalizability of the findings. Further research is needed to explore practical strategies to ensure that incidental processes of (provisional) reframing and institutional work contribute to longer-term shifts in field frames and more enduring/adaptive institutional logics, both within and across the boundaries of organizations and sectors. Additionally, there is some observational evidence that suggests that participants’ engagement in the CARE project may lead to spillover effects, fostering a more critical mindset in their everyday practices. Future research should focus on systemic investigations that explore how to sustain processes of institutional work and reframing, ensuring their contribution to longer-term changes.

In conclusion, this study has observed the complex interplay of diverse institutional logics within a public organization. It has demonstrated the potential uses and limitations of design-based practices in facilitating institutional change. While the methods employed proved effective in enabling individuals to challenge dominant logics and frames, continued efforts are needed to ensure that emerging logics are internalized and integrated into organizational practices and governance structures, to develop more resilient and adaptive public organizations.
